# The Medico-Psychological Association and Their Nurses

**Published:** 1897-01-02

**Authors:** 


### THE MEDICO-PSYCHOLOGICAL ASSOCIATION AND THEIR NURSES.

Some years ago the Medico Psychological Association began to advocate the training of the raw material forming the nursing staffs in asylums. The committee appointed to consider the question recommended a two years' training, during which at least twelve lectures by a medical officer must be attended. The subjects on which instruction was to be given were "limited to the ordinary requirements of nursing of, and attendance on, insane patients, combined with instruction in the general features of mental disease, together with general ideas of bodily structure and function, sufficient to enable nurses under training to understand such instruction, and to qualify them to render 'first aid,' especially in the case of accident or injury that may arise specially in asylums Anything beyond this is considered to be outside the scope of the present inquiry." The candidates after passing an examination receive a certificate of proficiency from the Association, and a register of the successful candidates is kept.

A friendly rivalry among superintendents has led to a great number of attendants and nurses gaining the certificate. The Association is now beginning to face a difficulty which was foreseen by all interested in nursing. The trained hospital nurse objects to these medico psychological nurses being ranked as on an equality with herself. While holding that the mental nurse and the hospital nurse do not stand quite on the same platform, and that it is not easy to compare them, we are disposed to sympathise with the latter. After a study of the report of the committee appointed to consider the question, and with some knowledge of the working of an asylum, we are reluctantly forced to believe that while some of the men and women holding these certificates are perfectly well equipped for their work there must be many who have little practical acquaintance with some of the most important and every-day duties of a nurse. In these cases the certificates can have little more value than the St. John's first aid and nursing certificates.

An asylum with one hundred nurses and attendants will have twenty or, may be, twenty-five of these in the sick wards. Now, without some special arrangement, which is very rarely done, how can the remaining seventy-five gain anything more than a theoretical knowledge of the work of a sick room. The knowledge can only be obtained by cramming from a text book.

The committee report that certificate given by a single asylum can only be a testimonial, and cannot have the value attaching to the document issued by the Association. Hospitals, however, train their own nurses and give their own certificates. The value of these certificates depends upon the reputation of the institution from which they come, and the authorities, mindful of this reputation, are not likely to award them carelessly. The committee hope to see the day when all responsible nursing posts in asylums will be barred against those who do not hold the medico psychological certificate. In the interests of the insane we trust that day is far distant Improvement lies not in that direction, but in the free competition of institutions which gain or lose credit according to the standard of their nursing.

Every mental nurse should pass a portion of her training in the sick wards. A better system, perhaps, would be that they should pass through the nearest hospital. This would do much to bring hospital and asylum more into touch. Perhaps we might venture to suggest also that questions which arise in the treatment of the insane might be more profitably discussed at meetings of the British Medical Association than at those of the Medico-Psychological. It is well that subjects should be looked at sometimes from a new point of view. The papers worth preserving would then not be lost to the busy practitioner.

This need not interfere with the social value of the Medico-Psychological meetings, and would leave its members free to discuss the pension question and administrative details; perhaps, in time, with the assistance of delegates from the nursing staff. The holders of these certificates will one day claim to be heard, and may even knock loudly at the sacred portals of the Council chamber.

